# Biological Activities of *Heteropyxis natalensis* Against Micro-Organisms Involved in Oral Infections

**DOI:** 10.3389/fphar.2018.00291

**Published:** 2018-04-10

**Authors:** Cynthia J. Henley-Smith, Francien S. Botha, Ahmed A. Hussein, Mpumelelo Nkomo, Debra Meyer, Namrita Lall

**Affiliations:** ^1^Department of Plant and Soil Science, University of Pretoria, Pretoria, South Africa; ^2^Paraclinical Sciences, Faculty of Veterinary Sciences, University of Pretoria, Pretoria, South Africa; ^3^Chemistry Department, Cape Peninsula University of Technology, Bellville Campus, Cape Town, South Africa; ^4^Von Seidels Intellectual Property Attorneys, Cape Town, South Africa; ^5^Faculty of Science, University of Johannesburg, Johannesburg, South Africa

**Keywords:** *Heteropyxis natalensis*, *Actinomyces israelii*, antibacterial, compound isolation, anti-inflammatory

## Abstract

The use of complementary and alternative medicine from plants in South Africa, as in the rest of the world, continues to increase. *Heteropyxis natalensis*, known as the Lavender tree, is indigenous to South Africa and is traditionally used for oral care. The ethanolic extract, of the leaves and twigs, of *H. natalensis* was investigated for antimicrobial activity against selected oral microorganisms. *Actinomyces israelii* was found to be the most sensitive oral microorganism to the extract, with a minimum inhibitory concentration (MIC) of 0.88 mg/ml and an MIC of 2.6 mg/ml against *Streptococcus mutans*. Five known compounds were identified from the ethanolic extract of *H. natalensis*. The compounds were identified as aurentiacin A (1), cardamomin (2), 5-hydroxy-7-methoxy-6-methylflavanone (3), quercetin (4) and 3,5,7-trihydroxyflavan (5). The MICs of the compounds 1 and 4 were found to be 0.06 mg/ml and 1 mg/ml, respectively, against *A. israelii*. The cytotoxicity, acute and sub-acute toxicity in pre-clinical studies were also determined for *H. natalensis.* The extract showed moderate cytotoxicity (35.56 ± 0.16 μg/ml) on human monocyte cells. The acute and sub-acute toxicity analysis of *H. natalensis* indicated the NOEL (no-observed-effect level) at 200 mg/kg. Interleukin-8 (IL-8) is a chemokine that stimulates the recruitment of leukocytes. A significant reduction of IL-8 production by macrophage cells was observed when exposed to the extract of *H. natalensis*. It is possible that *H. natalensis* can prevent excessive tissue damage in periodontal diseases through its reduction of inflammation. Enzymatic bioanalysis of lactic and acetic acid production from *Streptococcus mutans* and *Lactobacillus paracasei* was done. A reduction in the acid production from each bacterium was observed on exposure to the extract of *H. natalensis*. Consequently, this increased the pH, which could possibly reduce the demineralization of enamel which may help prevent the formation of dental caries. In addition the extract may be considered for preventing periodontal diseases.

## Introduction

*Heteropyxis natalensis* Harv., belonging to the family Heteropyxidaceae, is commonly known as the Lavender Tree. It is distributed in the Limpopo, Gauteng, and KwaZulu-Natal provinces of South Africa (Van Wyk and Van Wyk, 1997). It was listed by Van Wyk (2011), as an “indigenous South African plant species of historic, current or potential importance in the formulation of commercial medicine.” Decoctions of the leaves and twigs of *H. natalensis* are traditionally used by Venda and Zulu communities as an oral rinse for toothache and for oral and gum infections (Van Wyk and Gericke, 2000).

Several mouth rinses have been developed as agents for the prevention of dental caries, but dental caries is still a major factor in tooth loss, worldwide. Therefore, development of new preventive agents for dental caries is needed (Yu et al., 2007). Natural products, either pure compounds or standardized plant extracts, provide unlimited opportunities for novel and suitable additives and drugs because of their unmatched range of chemical diversities (Klančnik et al., 2010).

This study investigated several biological activities of *H. natalensis* related to the human oral cavity. These investigations included: antimicrobial activity of *H. natalensis* against four pathogenic oral microorganisms, *Actinomyces israelii*, *Streptococcus mutans*, *Prevotella intermedia*, *Candida albicans*, and against *Lactobacillus paracasei*, a commensal bacterium essential in plaque prevention. Commensal bacteria should preferably not be affected by any treatment to help ensure a healthy immune system is maintained and to prevent opportunistic microorganisms taking a hold of the environment. When bacteria cause an infection, pro-inflammatory cytokines, such as interleukin-8 (IL-8), are released from cells and can cause tissue destruction, such as gum loss, through excessive inflammatory responses. Reduction of the release of pro-inflammatory cytokines may prevent excessive tissue damage in periodontal diseases, therefore the effect of extract on the release of cytokines was analyzed. Enzymatic bioanalysis of lactic and acetic acid production from the acid producing bacteria, *S. mutans* and *L. paracasei* was investigated on exposure to the plant extract. Isolation of compounds using bioassay guided fractionation for the identification of bioactive(s) from the plant was conducted. The extract’s cytotoxicity, acute and sub-acute toxicity in mice were investigated to analyze the safety of the plant extract. This holistic approach covers the myriad of properties essential for an effective oral care pharmaceutical and/or alternative.

## Materials and Methods

### Plant Collection, Authentication, and Extract Preparation

Aerial plant parts, comprising the leaves and twigs of *H. natalensis* were collected from the University of Pretoria’s experimental farm. Voucher specimens were prepared and identified at the H.G.W.J. Schwelcherdt Herbarium (PRU), University of Pretoria (PRU 096405). Plant material was air dried at room temperature (25°C), and ground to a homogeneous blend using a Janke and Kunkel (IKA Labortechnik, Germany) grinder. The homogeneous material was extracted with 96% ethanol [Merck Chemicals (Pty) Ltd., Wadeville, South Africa] under pressure (100 bar), and regulated temperature of 50°C using a BUCHI Speed Extractor, E-916 (BUCHI Labortechnik AG, Switzerland). The solvent was evaporated at low boiling point in a Genevac, EZ-2 plus, after which all the extracts were subjected to various biological tests.

### Antimicrobial Activity

#### Microbial Strains

The microorganisms used in this study included *Actinomyces israelii* (ATCC 10049), *Prevotella intermedia* (ATCC 25611), *Streptococcus mutans* (ATCC 25175), *Lactobacillus paracasei* (oral clinical strain A54), *Candida albicans* (ATCC 10231), and a strain of *Candida albicans* resistant to the drugs; imidazole and polyene (1051604). The bacteria were grown on Casein-peptone Soymeal-peptone (CASO) Agar [Merck Chemicals (Pty) Ltd., Wadeville, South Africa] enriched with 1% sucrose [Merck Chemicals (Pty) Ltd., Wadeville, South Africa] under anaerobic conditions in an anaerobic jar with Anaerocult^®^ A [Merck Chemicals (Pty) Ltd., Wadeville, South Africa], at 37°C for 48 h. *Candida albicans* was grown on Sabouraud Dextrose 4% Agar (SDA) [Merck Chemicals (Pty) Ltd., Wadeville, South Africa], at 37°C for 48 h. Sub-culturing was done every second week. Inoculum was prepared by suspending microbial test organisms in enriched CASO broth [Merck Chemicals (Pty) Ltd.] for the bacteria and Sabouraud Dextrose 4% broth [Merck Chemicals (Pty) Ltd.] for *Candida* until turbidity was found to be compatible with McFarland Standard 1 [Merck Chemicals (Pty) Ltd., Wadeville, South Africa] (McFarland, 1907).

#### Determination of Minimum Inhibitory Concentration (MIC) and Minimum Bactericidal Concentration (MBC)

The microdilution technique using 96-well micro-plates, as described by Eloff (1998) was used to obtain the MIC and MBC values of the crude extracts against the microorganisms under study. The extract was dissolved in 20% dimethyl sulfoxide (DMSO) (Merck Chemicals (Pty) Ltd.), and serially diluted in broth (enriched for *A. israelii* and *S. mutans*) for the bacteria and sterile water for *C. albicans*; in the 96-well plate adding 48 h old microorganisms grown at 37°C. The final concentration of extracts ranged from 0.10 to 12.5 mg/ml and the positive control, 5% chlorhexidine gluconate (CHX) (Dental Warehouse, Sandton, South Africa), ranged from 3.8 × 10^-4^ to 12.5 mg/ml for each microorganism tested. Amphotericin B (Davis Diagnostics, Gauteng) an established antifungal drug, ranging from 0.2 mg/ml to 1.5 × 10^-3^ mg/ml, was included for the *Candida* assays. The highest concentration of the solvent DMSO (5%) was found to be non-toxic to the microorganisms tested. The plates inoculated with bacteria were incubated at 37°C, under anaerobic conditions and the plates inoculated with *C. albicans* under aerobic conditions for 24 h.

To indicate bacterial growth, 20 μl (0.2 mg/ml) of INT, or PrestoBlue was added to micro-plate wells and incubated at 37°C, under anaerobic conditions, for 20–60 min until a color change occurred. The MIC was defined as the lowest concentration that inhibited the color change of INT or PrestoBlue. The MBC was determined by adding 50 μl of the suspensions from the wells, which did not show any growth after incubation during MIC assays, to 150 μl of fresh broth. These suspensions were re-incubated at 37°C for 24 h. The MBC was determined as the lowest concentration of extract which inhibited 100% growth of microorganisms (Cohen et al., 1998; Lall et al., 2013). All test samples, as well as the controls, were done in triplicate on the 96-well microtiter plates in three independent experiments.

### Determination of Cytotoxicity

Human monocyte (U937) cells (ATCC: CRL 1593), Kidney epithelial cells of the African Green Monkey (Vero) and Human laryngeal epidermoid carcinoma (HEp-2) cells were used for testing the samples for cytotoxicity (Sigma-Aldrich, 2003). The cells were maintained in RPMI (Roswell Park Memorial Institute) medium supplemented with 10% fetal bovine serum (FBS) and 1% antibiotics (100 U/ml penicillin, 100 μg/ml streptomycin) and 250 μg/ml fungizone [supplied by Highveld Biological (Pty) Ltd., Johannesburg, South Africa]. The cells were grown at 37°C in a humidified incubator set at 5% CO_2_. Cells were sub-cultured by centrifuging at 980 rpm for 5 min and re-suspending in fresh medium. The method described by Lall et al. (2013), was used to perform the assay. One hundred microliters of U937 cells (1 × 10^5^ cells/ml) were added to 96-well microtiter-plates and incubated for 24 h to allow the cells to differentiate and adhere as macrophages to the bottom of the plate. Stock solutions of the ethanol extract of *H. natalensis* were prepared in DMSO [Merck Chemicals (Pty) Ltd.]. The stock solutions were serially diluted in supplemented RPMI medium. The final concentration of *H. natalensis* ranged from 3.125 to 400 μg/ml. The highest concentration of DMSO (2%) was found to be non-toxic to the cells. The dilution series of the samples were added to the microtiter-plate and incubated for a further 72 h. Thereafter 20 μl PrestoBlue (Invitrogen Corporation, San Diego, CA, United States) was added to the plates of U937 cells away from direct light and the cells were incubated for 2–3 h. The Vero and HEp-2 cells cytotoxicity was measured by the XTT (Sodium 3’-[1-(phenyl amino-carbonyl)-3,4-tetrazolim]-bis-[4 methoxy-6-nitro] benzene sulfonic acid hydrate) method using the cell proliferation kit II (Roche Diagnostics GmbH). The positive drug control Actinomycin D (Sigma-Aldrich, South Africa), at a final concentration range of 3.9 × 10^-4^ – 0.78 μg/ml, was included in the assay. After incubation, the absorbance of the color complex was spectrophotometrically quantified using an enzyme-linked immunosorbent assay (ELISA) plate reader (BIO-TEK Power-Wave XS, Weltevreden Park, South Africa), which measured the optical density at 570 nm with a reference wavelength of 600 nm for the U937 cells and at 490 nm with a reference wavelength of 690 nm for the Vero and HEp-2 cells. The assays were carried out in triplicate. The IC_50_ values were determined using GraphPad Prism version 4.03.

### Acute and Sub-acute Toxicity in a Mouse Model

The acute and sub-acute toxicity of the dried 96% ethanolic extract of *H. natalensis* in mice was conducted by pathologists in the Faculty of Veterinary Science, University of Pretoria in Pretoria. The Animal Ethics Committee approved the investigation conducted on 64 mice by Professor V. Naidoo; (project number V065-14). A 2-week toxicity study with a once a day oral gavage dosing of 0 mg/kg, 50 mg/kg, 100 mg/kg, and 200 mg/kg, according to the OECD guideline 408 (repeated dose 90-day oral toxicity study in rodents) was conducted. Forty inbred CD1 mice, the recommended species for routine toxicity testing, were divided into four groups of equal sexes. Parameters investigated include: animal weights, feed intake, clinical signs of toxicity, gross necropsy, full histopathology, full hematology and selected clinical pathology.

### Reduction of Pro-inflammatory Response

#### Preparation of Heat-Killed Microorganisms

When bacteria cause an infection, pro-inflammatory cytokines are released from cells and may cause tissue destruction through excessive inflammatory responses. IL-8 is a chemokine, which is defined as a family of low-molecular weight pro-inflammatory cytokines that stimulate the recruitment of leukocytes. As *A. israelii* does not appear to stimulate significant production of IL-8; *P. intermedia* and *C. albicans* were selected for pro-inflammatory evaluation of ethanol extracts of *H. natalensis.* These microorganisms were cultured in their respective broths for 72 h at 37°C under anaerobic (*P. intermedia*) and aerobic conditions (*C. albicans*). The log-phase microbial culture was harvested, washed three times with phosphate buffer saline (PBS) and incubated at 80°C for 20 min to kill the microorganism. The heat-killed microorganisms were stored at 4°C until use (Jiang et al., 1996; Tsai et al., 2010).

To determine whether the plant extract reduced the pro-inflammatory response of *P. intermedia* or *C. albicans* to the U937 cells, time-dependent studies were conducted. Firstly, *H. natalensis* extract, at 50% inhibitory concentration (IC_50_) of 35.56 μg/ml and sub-cytokine value of 17.78 μg/ml (half the inhibitory concentration), was added separately to the prepared macrophages and 1 h later *P. intermedia* was added (Treatment 1). Secondly, the plant extract and *P. intermedia* were added to the macrophages together (Treatment 2) and lastly, *P. intermedia* was added to the macrophages with the plant extract being added 1 h later (Treatment 3). This process was repeated for the synergistic combinations for *P. intermedia* and *C. albicans*.

U937 cells were seeded at 1 × 10^6^ cells/ml in 24-well plates with serum free medium, and were stimulated with LPS or heat killed *P. intermedia* or *C. albicans* (wet weight 100 μg/ml) alone or in combination with the ethanolic extract of *H. natalensis* or the specified synergistic combination for each microorganism for 24 h incubation. Cell-free supernatants were collected and the concentration of IL-8 was analyzed with the enzyme-linked immunosorbent assay (ELISA) kit (Pharmigen, OptEIA Human IL-8 Set, catalog no. 555244) obtained from BD Bioscience (Tsai et al., 2010).

### Acid Analysis

The L-Lactic test (Roche, R-Biopharm, Enzymatic BioAnalysis, catalog no. 10139084035) and Acetic acid test (Roche, R-Biopharm, Enzymatic BioAnalysis, catalog no. 101480261035) were conducted as described in the kits. Analysis of lactic and acetic acid from *S. mutans* and *L. paracasei* were carried out for the samples after 24 h incubation with the *H. natalensis* plant extract. The samples for lactic acid were collected at MIC and sub-minimum inhibitory concentrations (sub-MIC) which are half the MIC values; except for acetic acid which is not strongly produced, therefore only MIC samples were collected. As described in the kit manuals, the samples for lactic acid had to be adjusted to a pH between 8 and 10, and for acetic acid between, 8 and 9. Trial runs of each kit showed that the samples had to be suitably diluted to remain within the accuracy range of the enzymatic catalysis. Therefore, for both lactic and acetic acid, 1 in 10 dilutions were made from the samples; these were then adjusted to their correct pH. Undiluted samples were also monitored for pH to determine whether the extract of *H. natalensis* and the synergistic combination for *S. mutans* as described in Henley-Smith et al. (2014), could increase the pH even in the presence of acid producing bacteria.

### Purification of Active Compounds

The dried ethanolic extract of *H. natalensis* (60 g) was subjected to fractionation on a silica column (10 cm × 70 cm) using a gradient of hexane:ethyl acetate of increasing polarity (0% to 100% ethyl acetate) as eluent. Twenty-nine fractions were collected and those with similar thin layer chromatography (TLC) profiles were combined together. TLC plates were developed using hexane:ethyl acetate (7:3); hexane:ethyl acetate (8:2); dichloromethane:methanol (99.5:0.5) and dichloromethane:methanol (99:1) as eluent. Acidic vanillin (0.34% vanillin in 3.5% sulfuric acid in methanol) was used for detection. Thirteen major fractions (1B to 13B) were obtained and tested for antibacterial activity against *A. israelii*.

**FIGURE 1:**
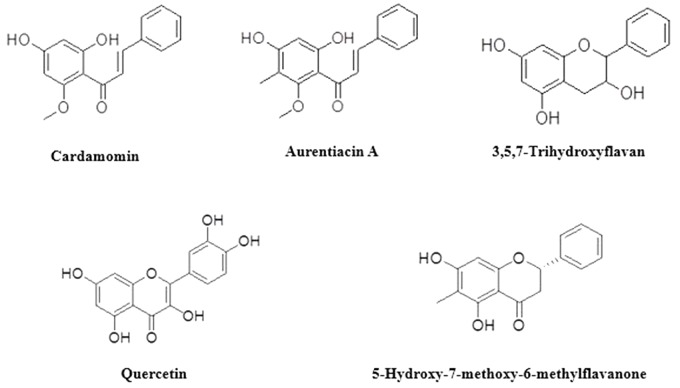
Compounds identified from the fractions.

Based on the antibacterial results and preliminary TLC plates; Fractions 7B (2 g), 9B (3 g), 11B (0.8 mg) and 12B (1 g) were chromatographed separately using Sephadex columns (Sigma-Aldrich, South Africa). Fraction 7B was chromatographed using dichloromethane:methane (99.5:0.5). Sixty-four subfractions were collected, spotted on TLC plates and developed in dichloromethane:methane (99:1). Pure compound **1** (aurentiacin A; 87 mg, 0.150%) (Supplementary Figure 1) was obtained. Fraction 9B was chromatographed using 96% ethanol. Ninety-one sub-fractions were collected, spotted on TLC plates and developed in dichloromethane:methane (99.5:0.5). Pure compounds **2** (cardamomin; 2 mg, 0,003%) (Supplementary Figure 2) and **3** (5-hydroxy-7-methoxy-6-methylflavanone, 22 mg, 0,037%) (Supplementary Figure 3) were obtained. Fraction 11B was chromatographed using dichloromethane:methane (99:1). Fifty-five sub-fractions were collected, spotted on TLC plates and developed in dichloromethane:methane (99:1). Pure compound **4** (quercetin, 75 mg, 0.130%) (Supplementary Figure 4) was obtained. Fraction 12B was chromatographed using dichloromethane:methane (99:1). Seventy-one sub-fractions were collected, spotted on TLC plates and developed in dichloromethane:methane (99:1). Pure compound **5** (3,5,7-trihydroxyflavan, 7 mg, 0.012%) (Supplementary Figure 5) was obtained. **Figure 1** shows the compounds identified. The identified compounds were then tested for antimicrobial activity.

## Results and Discussion

The ethanol extract of *H. natalensis* exhibited the best MIC value against *A. israelii* at 0.88 mg/ml (**Table 1**). Folk medicine from Brazil, used to treat oral diseases, was evaluated against *P. intermedia* and *S. mutans* by Alviano et al. (2008). Their study utilized *Cocos nucifera*, known to contain catechin and epicatechin; the inner bark of *Ziziphus joazeiro*; leaves of *Caesalpinia pyramidalis* and a 50% ethanolic extract from *Aristolochia cymbifera.* Our investigation results for *S. mutans* (2.6 mg/ml) and lactobacilli (9.38 mg/ml) correspond with the results attained in the investigation by Alviano et al. (2008). However, the MIC for *P. intermedia* was observed to be higher (12.5 mg/ml) as compared to the MIC of the folk medicine from Brazil. The bacterial suspension used by Alviano et al. (2008) was standardized to a 0.5 McFarland Standard as compared to the one used in the present study (1.0 McFarland Standard) which is almost double the bacterial suspension.

**Table 1:** The minimum inhibitory concentration (MIC) and minimum bactericidal concentration (MBC) of the ethanolic extract of *H. natalensis* on oral microorganisms.

Plant extract	Microbial activity											
	
	MIC (mg/ml)							MBC (mg/ml)				
		
	Gram +ve			Gram -ve	Yeast		Gram +ve			Gram -ve	Yeast	
					
	A.i	S.m	L.p	P.i	C.a	C.a (res)	A.i	S.m	L.p	P.i	C.a	C.a (res)
*Heteropyxis natalensis*	0.88	2.60	9.38	12.5	8.33	12.5	3.32	9.38	12.5	>12.5	10.42	>12.5
Positive controls	0.033^a^	0.061^a^	1.2 × 10^-3a^	0.26^a^	0.352^a^	0.26^a^	0.039^a^	1.2 × 10^-3^	2.4 × 10^-3a^	1.042^a^	0.293^ab^	0.293^a^
					>0.2^b^	0.013^b^						0.013^b^


*^*a*^Chlorhexidine; ^*b*^Amphotericin B; A.i, *Actinomyces israelii*; S.m, *Streptococcus mutans*; L.p, *Lactobacillus paracasei*; P.i, *Prevotella intermedia*; C.a, *Candida albicans*; C.a (res), Polyene and azole resistant *C. albicans.**

Several other medicinal plants have been investigated for activities against oral pathogens. The aqueous and methanol extract of the bark of *Piptadeniastrum africanum*, traditionally used for abdominal pain in Cameroon, was tested for inhibitory activity against *S. mutans* (ATCC 25175). Twofold serial broth macro-dilutions were used to determine the MIC with an average inoculum of 10^7^ CFU/ml. An MIC of 1 mg/ml and 0.7 mg/ml was obtained for the aqueous and methanol extracts, respectively (Brusotti et al., 2013). *Curcuma longa* (turmeric) has been used in India to maintain oral hygiene. A methanol extract of *C. longa* was tested against *S. mutans* (UA159). The MIC was obtained at 1 mg/ml at a 1-1.5 × 10^6^ CFU/ml (Pandit et al., 2011). The MIC values of the methanol extracts of *P. africanum* and *C. longa* are lower when compared to the activity of the ethanol extract of *H. natalensis* (2.60 mg/ml) against *S. mutans*. However, the bacterial suspension must be taken into consideration as a McFarland Standard 1.0 had a bacterial suspension density of 3.0 × 10^8^ CFU/ml for *S. mutans* in the present investigation.

For the anti-yeast activity of *H. natalensis*, the extract was found to be relatively active with an MIC of 8.33 mg/ml obtained against a yeast suspension of 4 × 10^7^ cells/ml, as compared to a study by Motsei et al. (2003), where no anti-candidal activity was obtained for their plants tested. As *H. natalensis* is not used traditionally for candidal infections, it compares well with plants cogitated to anti-candidal properties. An acetone extract of *Dodonaea viscosa*, another plant used in transitional oral care, was tested for antifungal activity against *C. albicans* (ATCC 90028) inocula with 10^6^-10^7^ cells/ml. The MIC ranged from 6.25 to 25 mg/ml and correlated with our findings for *H. natalensis* (Patel and Coogan, 2008).

Bioassay guided isolation revealed the presence of five compounds in the extract of *H. natalensis*. These were, cardamomin, aurentiacin A, 5-hydroxy-7-methoxy-6-methylflavanone, 3,5,7-trihydroxyflavan and quercetin (**Figure 1**). The MICs and MBCs of the isolated compounds against *A. israelii* are shown in **Table 2**. These revealed good inhibitor strength (below 1.5 mg/ml) despite the lack of congruency on measuring strength as per the classification used by Nkomo et al. (2014). The crude extract exhibited an MIC value of 1.56 mg/ml and aurentiacin A showed the best activity at 0.0625 mg/ml against *A. israelii*.

**Table 2:** The minimum inhibitory concentration (MIC) and minimum bactericidal concentration (MBC) of the major fractions and isolated compounds from *H. natalensis* against *Actinomyces israelii*.

Isolated compounds	MIC (mg/ml)	MBC (mg/ml)
*Heteropyxis natalensis*	1.5625	1.5625
5-Hydroxy-7-methoxy-6-methylflavanone (**3**)	–	–
Aurentiacin A (**1**)	0.0625	0.0625
Cardamomin (**2**)	>1	NA^a^
3,5,7-Trihydroxyflavan (**5**)	>1	NA
Quercetin (**4**)	1	1
Positive control	0.024*^b^*	0.024*^b^*
**Fractions**		
1B	>12.5	NA^a^
2B	>12.5	NA
3B	>12.5	NA
4B	>12.5	NA
5B	9.375	9.38
6B	9.375	9.38
7B	0.83	1.61
8B	0.83	2.34
9B	1.56	2.34
10B	2.21	8.20
11B	4.25	12.5
12B	12.5	NA
13B	1.82	5.47
Positive control^b^	0.018311	0.048828


*^*a*^NA, not active; ^*b*^Chlorhexidine.*

The leaves of *H. natalensis* were examined for their phytochemical potential. A dichloromethane extract was eluted through silica gel from which a yellowish crystalline compound was obtained. It was determined to be a chalcone isomer of aurentiacin A and triangularin previously isolated from *Myrica serrate* and *Pityrogramma triangularis*. Spectroscopic data established the compound to be (*E*)-1-(2’,4’-dihy-roxy, 5’-methoxy, 3’-methylphenyl)-3-phenylprop-2-en-1-one (Adesanwo et al., 2009). Preliminary results from a study showed that (2E)-2-[(2E)-1-hydroxy-3-phenylprop-2- en-1-ylidene]-5-methoxy-6,6-dimethylcyclohex-4-ene-1,3-dione, commonly known as ceroptin was also present in the leaf extract of *H. natalensis* (Shode et al., 2005).

The twigs and roots of *H. natalensis* were also investigated for phytochemicals. A CH_2_Cl_2_ extract of the twigs yielded 2’,4’-dihydroxy-6’-methoxy-3’,5’-dimethylchalcone; 3’,4’,5’-tri-*O*-methyl-3,4-methylenedioxyellagic acid and lupane derivatives betulinic acid. Hexane extracts of the twigs yielded lupenone and lupeol; and from the roots 3β-hydroxylup-20(29)-en-28-al and sitost-4-en-3-one (Mohammed et al., 2009).

As the use of any plant derived medicine invariably requires to be safe and not harmful to the body in any form, cytotoxicity analysis of extracts is imperative. Cytotoxicity tests revealed a general trend with the extract of *H. natalensis*, indicating a decrease in viability of U937 cells as the concentration of the samples tested increased. Actinomycin D was used as a viability control and demonstrated the marked decrease in viability on U937 cells tested in a dose-dependent manner. The cytotoxicity of the extract was determined as a percentage of the positive control, which were cells grown in medium only. The extract of *H. natalensis* showed an IC_50_ of 35.56 ± 0.16 μg/ml 147 ± 0.150 μg/ml and 33.66 ± 0.04 μg/ml on macrophage U937 cells, Vero and HEp-2 cells, respectively. The positive control, Actinomycin D, showed IC_50_ values of 1.6 × 10^-3^ ± 2.8 × 10^-4^ μg/ml 8.5 × 10^-3^± 9.95 × 10 μg/ml and 0.06 ± 2.44 μg/ml on macrophage U937 cells, Vero and HEp-2 cells, respectively. Therefore, on Vero cells, the extract of *H. natalensis* might be potentially harmful but on HEp-2 and U937 cells *H. natalensis* could be potentially toxic.

From the results of the animal trials, based on the absence of significant clinical or pathological effects being seen in the sub-acute study, it could be concluded that *H. natalensis* is not overtly toxic at the evaluated doses. While numerous changes were seen in clinical pathology, none of the changes showed a dose response relationship, and in most cases the effect was limited to the low dose group compared to the control. Changes were seen in the lymphocyte counts for the females. This change was considered to be of negligible biological significance as it was still within reported strain reference intervals. The only other indication of toxicity was a change in absolute and relative liver weights for the male animals. For this parameter, the middle dose group average weight was significantly lower than the controls (*p* < 0.5), while the high dose group tended toward significance (*p* < 0.10). When this change was considered in conjunction with the other parameters of hepatotoxicity such as clinical pathology and histopathology, no general indication of hepatotoxicity was evident. As a result of the smaller liver weights, the no-observed-effect level (NOEL) was set at 50 mg/kg for males while the absence of concurrent clinical chemistry or histopathological changes expected for liver damage will result in a no-observed-adverse-effect level (NOAEL) of 200 mg/kg for males. For the combined group and females, the NOEL was set at 200 mg/kg. Other plants with similar antibacterial activities to *H. natalensis* have been tested for acute toxicities in mice. An aqueous extract of the husk fiber of *Cocos nucifera*, known to contain catechin and epicatechin, the inner bark of *Ziziphus joazeiro*, leaves of *Caesalpinia pyramidalis* and a 50% ethanolic extract from *Aristolochia cymbifera* were evaluated after different doses (1–5 g/kg) were administered orally to mice. The mice were observed after 14 days and no weight loss or lethal effects were noticed in the mice at the tested doses, and a lethal dose for 50% of the population (LD_50_) corresponded to 2 g/kg (Alviano et al., 2008).

**FIGURE 2:**
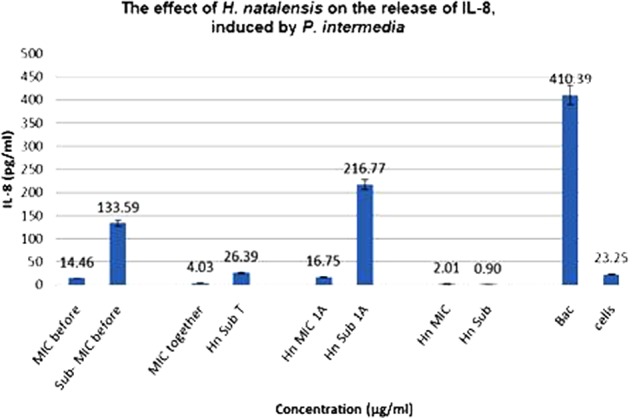
The effect of *Heteropyxis natalensis* on the release of IL-8, taken in a time-dependent manner at an IC_50_ and Sub-IC_50_ concentration; as compared to the controls, *Prevotella intermedia* only (Bac,) and cells only. (Treatment 1 at MIC = MIC before; Treatment 1 at Sub-MIC = Sub-MIC before; Treatment 2 at MIC = MIC together; Treatment 2 at Sub-MIC = Hn Sub T; Treatment 3 at MIC = Hn MIC 1 A; Treatment 3 at Sub-MIC = Hn Sub 1 A; *H. natalensis* at MIC = Hn MIC; *H. natalensis* at sub-MIC = Hn Sub).

The anti-inflammatory results for *H. natalensis* showed that higher concentrations of *H. natalensis* led to an increased suppression of the production of IL-8. All the treatments with *H. natalensis* at the IC_50_ concentration of 35.56 μg/ml suppressed the release of IL-8 by the cells, at least sixfold. The best results were obtained with Treatment 2, where the plant extract was added at the same time as the heat-killed *P. intermedia*. Even though the cells released some IL-8, when *H. natalensis* was added at the IC_50_ concentration it produced a reduction of IL-8. These results are also significant when comparing to the negative control of *P. intermedia* (410.39 pg/ml) (**Figure 2**).

**FIGURE 3:**
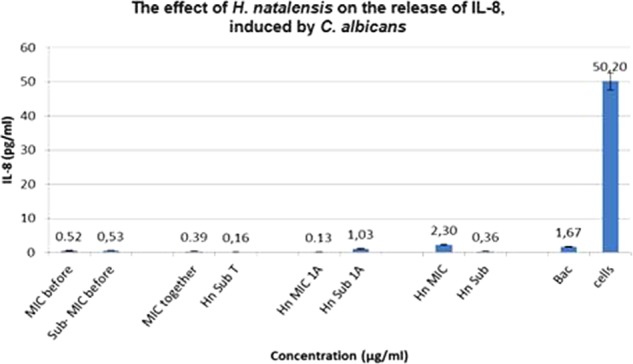
The effect of *Heteropyxis natalensis* on the release of IL-8, taken in a time-dependent manner at an IC_50_ and Sub-IC_50_ concentration; as compared to the controls, *Candida albicans* only (Bac) and cells only. (Treatment 1 at MIC = MIC before; Treatment 1 at Sub-MIC = Sub-MIC before; Treatment 2 at MIC = MIC together; Treatment 2 at Sub-MIC = Hn Sub T; Treatment 3 at MIC = Hn MIC 1 A; Treatment 3 at Sub-MIC = Hn Sub 1 A; *H. natalensis* at MIC = Hn MIC; *H. natalensis* at sub-MIC = Hn Sub).

Unlike *P. intermedia*, *C. albicans* did not induce a significant amount of IL-8, only 1.67 pg/ml. For Treatment 1, *H. natalensis* at an IC_50_ concentration of 35.56 μg/ml obtained virtually the same as the sub-IC_50_ concentration at 0.52 and 0.53 pg/ml, respectively. Treatment 3, where *C. albicans* was added 1 h before the extract, resulted in the biggest difference with IC_50_ concentration suppressing IL-8 release to 0.13 pg/ml while the sub-IC_50_ concentration could only suppress the IL-8 to 1.03 pg/ml (**Figure 3**).

In response to bacterial stimuli and commensal bacteria, epithelial cells are thought to produce various pro-inflammatory cytokines. However, this stimulus by bacteria appears to be quite specific as some oral epithelial cells do not release an increase in the amount of pro-inflammatory cytokines such as IL-8. This may be in response to how epithelial cells interact with commensal bacteria to ensure that these cells do not produce pro-inflammatory cytokines which may lead to tissue destruction through an excessive inflammatory response. Another possibility is that the dose of the bacterial ‘challenge’ to the cells may also play a role (Jiang et al., 1998; Uehara et al., 2007).

In assessing acid production by *S. mutans* and *L. paracasei*. Results showed that acetic acid was not produced in large quantities by either *S. mutans* or *L. paracasei* (**Table 3**). However, *H. natalensis* at an MIC of 9.38 mg/ml reduced the amount of acetic acid produced by *L. paracasei* (0.33 g/L) by almost half (0.19 g/L). The acetic acid production by *S. mutans* (0.26 g/L) was also reduced by the inhibitory concentration of *H. natalensis* (MIC of 2.6 mg/ml). *Lactobacillus paracasei*, produced a large quantity of L-lactic acid (3.47 g/L) (**Table 3**).

**Table 3:** Acetic acid and L-lactic acid production by *Streptococcus mutans* and *Lactobacillus paracasei* on exposure to ethanolic extract of *H. natalensis*.

L-Lactic acid production		*S. mutans*		*L. paracasei*
		
	pH	g acetic acid/l sample sol	pH	g acetic acid/l sample sol
*H. natalensis* – MIC	5.84	0.22	5.51	0.16
*H. natalensis* – Sub-MIC	5.78	0.21	5.73	0.11
Negative control	4.32	1.38	3.63	3.47
**Acetic acid production**				
*H. natalensis* – MIC	5.84	0.21	5.51	0.19
Negative control	4.32	0.26	3.63	0.33


*MIC, minimum inhibitory concentration; Sub-MIC, half the minimum inhibitory concentration; negative control, bacteria only.*

Green tea has been found to halt the production of lactic acid by inhibiting bacterial lactate dehydrogenase which may explain the latter activity (Song and Seong, 2007). The decrease in pH was significantly inhibited in *S. mutans* and *L. paracasei* with the presence of *H. natalensis* extract for each bacterium. Once the pH level falls below 5.0 to 5.5, enamel demineralization occurs (Loesche and Grossman, 2001). The *H. natalensis* extract at MIC and sub-MIC values appeared to prevent enamel demineralization with both bacterium.

*Heteropyxis natalensis* exhibited activity against the Gram-positive microorganisms, *A. israelii* and *S. mutans* and reduced activity against the Gram-negative bacteria, *P. intermedia*. The acute and sub-acute toxicity of *H. natalensis* was conducted by pathologists at the Faculty of Veterinary Science, University of Pretoria and results indicated that the NOEL was set at 200 mg/kg. It is possible that *H. natalensis* can prevent excessive tissue damage in periodontal diseases through its reduction of pro-inflammatory cytokine concentrations. *H. natalensis* exhibited the ability to reduce acid production by *S. mutans* and *L. paracasei*. Consequently, this increased the pH, possibly reducing the demineralization of enamel which should help prevent the formation of dental caries.

## Conclusion

The work reports for the first time *H. natalensis* activity against *A. israelii*, *L. paracasei*, as well as its *in vivo* acute and sub-acute toxicity. Five isolated compounds were identified for the first time from the ethanolic extract of *H. natalensis* leaves and twigs. The compounds were identified as aurentiacin A (**1**), cardamomin (**2**), 5-hydroxy-7-methoxy-6-methylflavanone (**3**), quercetin (**4**) and 3,5,7-trihydroxyflavan (**5**). The extract of H. natalensis was able to reduce the acid produced by the bacteria *S. mutans* and *L. paracasei*, which duly increased the pH. *H. natalensis* has the potential to reduce the growth and metabolic effects of cavity causing bacteria, while only moderately affecting commensal bacteria. The results indicated the possible use of *H. natalensis* to prevent excessive tissue damage in periodontal diseases through its reduction of pro-inflammation.

## Author Contributions

CH-S, NL, DM, FB, AH, and MN planned the experiments and/or wrote the manuscript. CH-S performed the experiments. CH-S, FB, and NL carried out the data analysis. NL and FB supervised all work. All authors critically revised and approved the final version of the manuscript.

## Conflict of Interest Statement

The authors declare that the research was conducted in the absence of any commercial or financial relationships that could be construed as a potential conflict of interest.
