# Supplementary figures and images for: Biological Activities of Heteropyxis natalensis Against Micro-Organisms Involved in Oral Infections
Source: Front Pharmacol. 2018 Apr 10;9:291. doi: 10.3389/fphar.2018.00291 (PMC5903190; doi:10.3389/fphar.2018.00291)

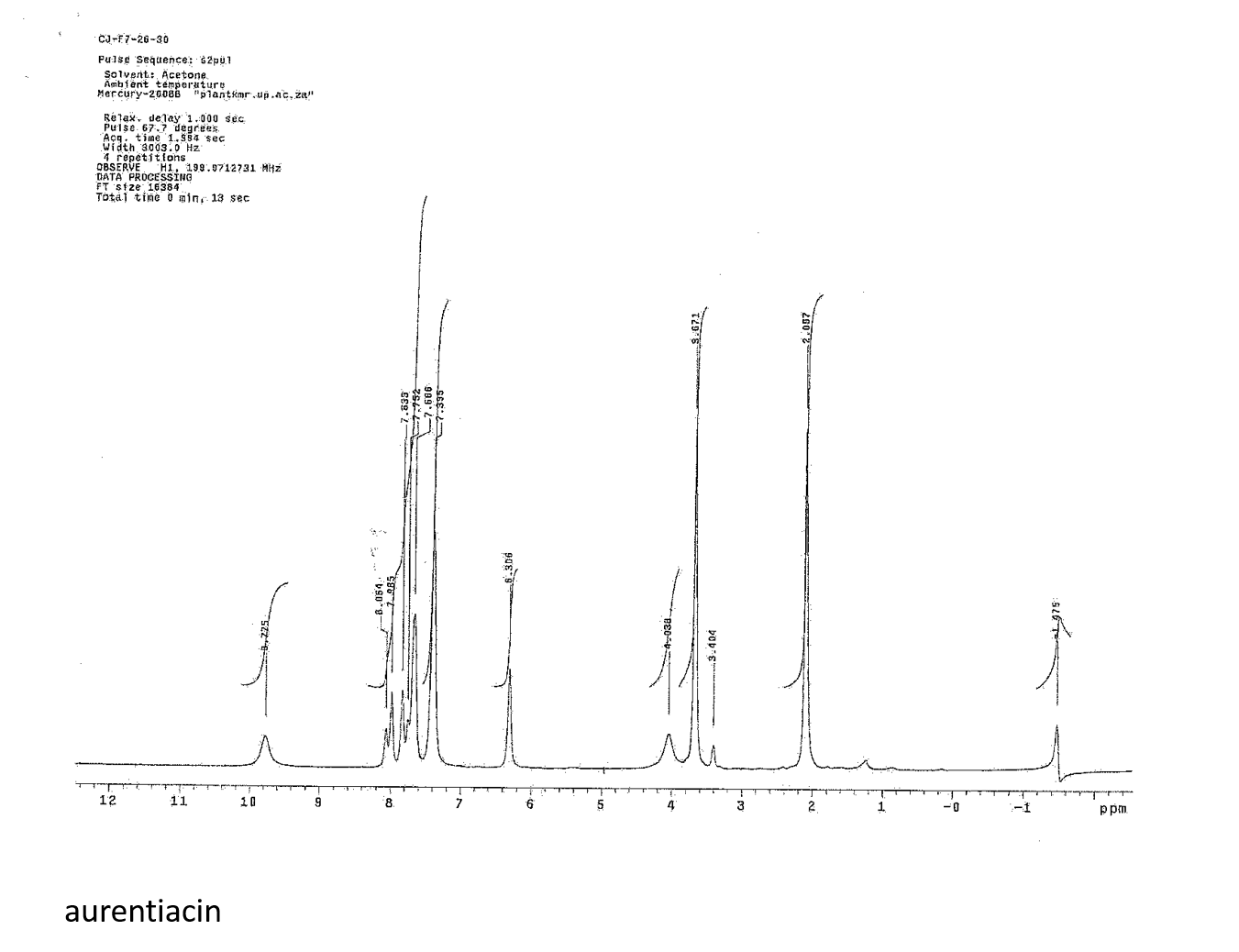

Supplement: Supplementary file 1 [file Image_1.tif]

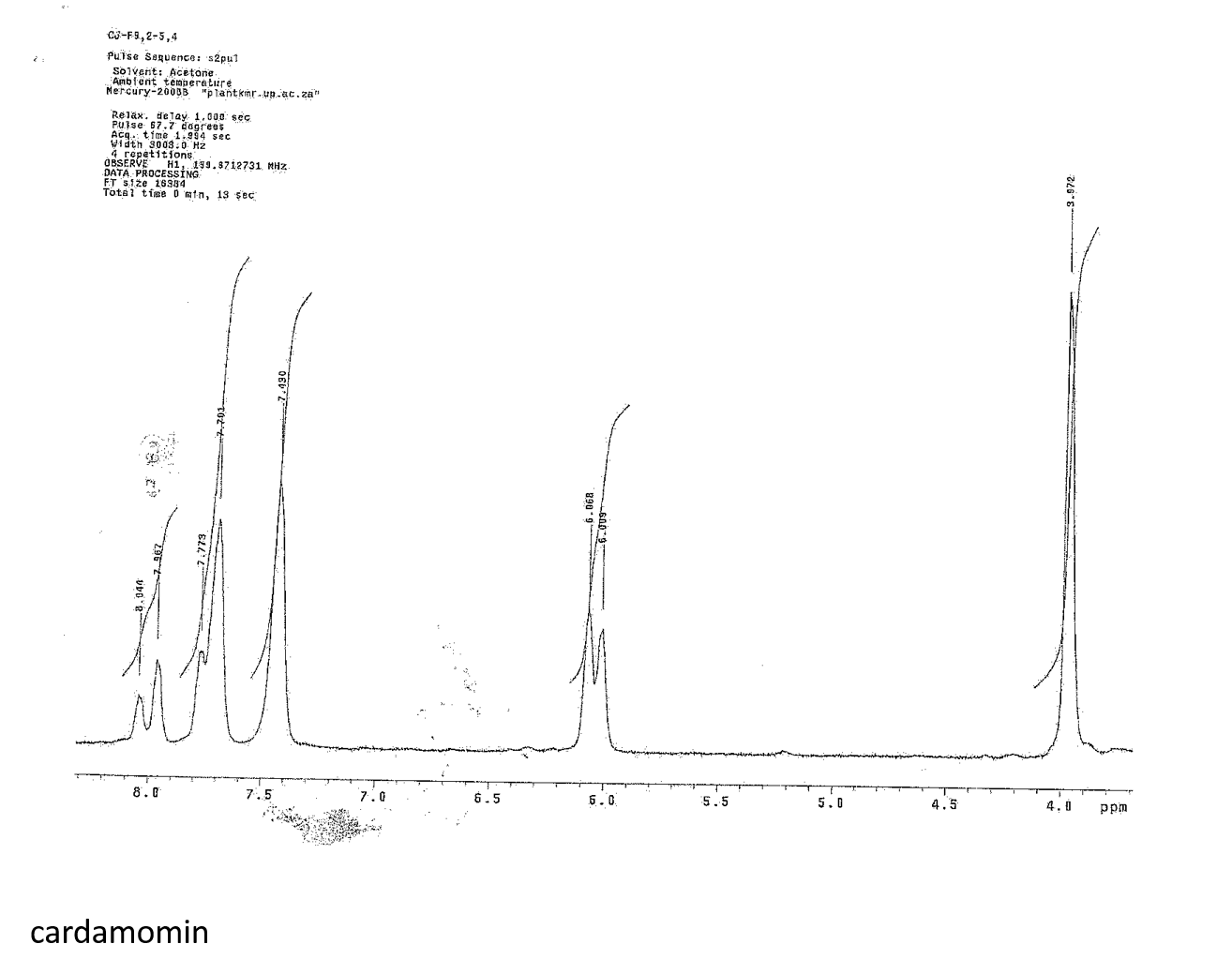

Supplement: Supplementary file 2 [file Image_2.tif]

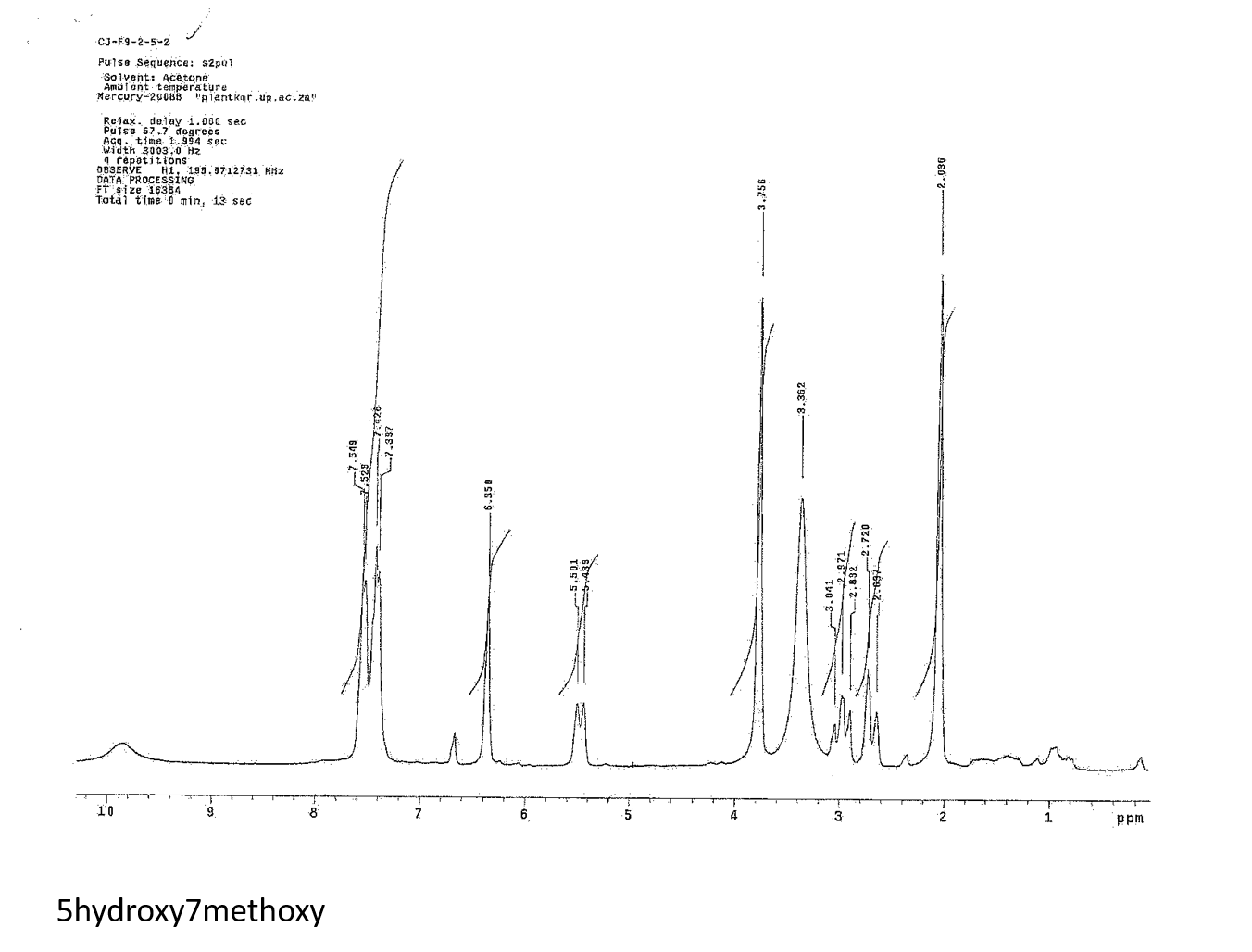

Supplement: Supplementary file 3 [file Image_3.tif]

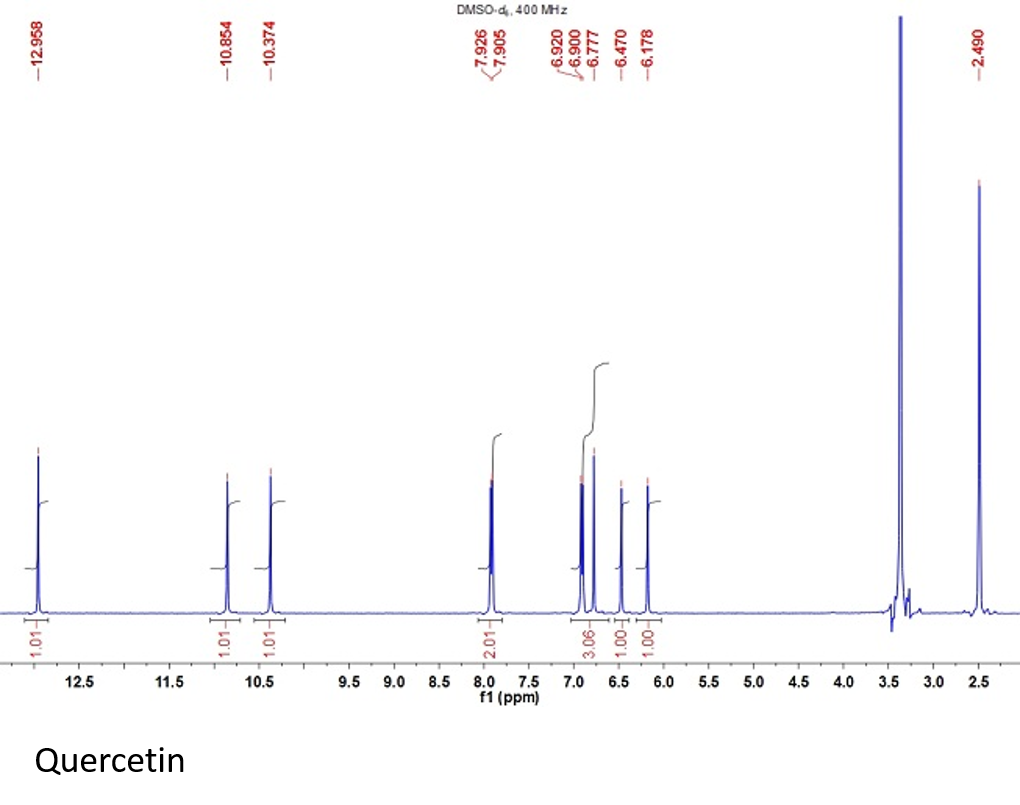

Supplement: Supplementary file 4 [file Image_4.tif]

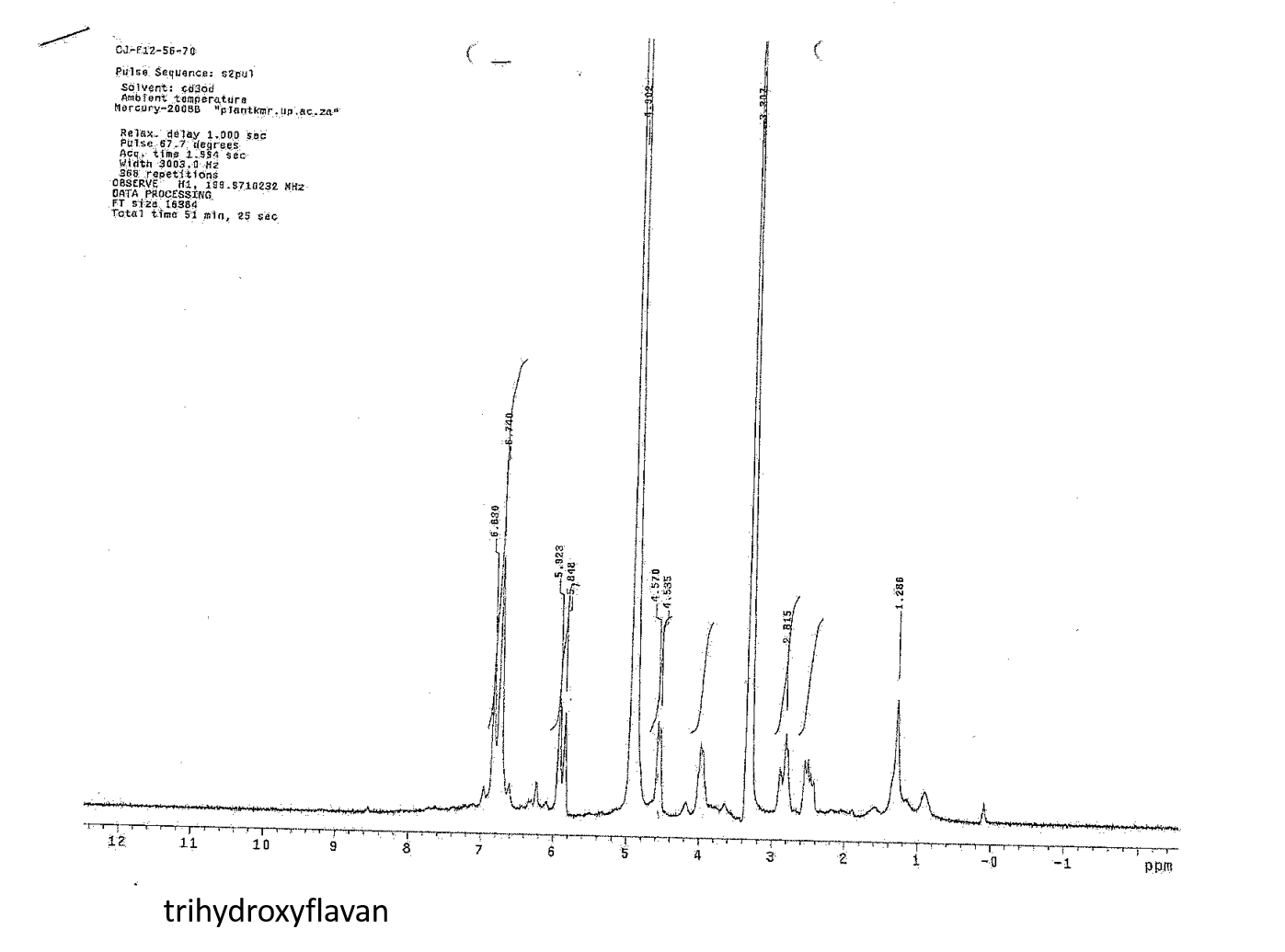

Supplement: Supplementary file 5 [file Image_5.tif]
